# Autism Spectrum Disorder and Mental Health Problems: Patterns of Difficulties and Longitudinal Trajectories in a Population-Based Twin Sample

**DOI:** 10.1007/s10803-021-05006-8

**Published:** 2021-04-17

**Authors:** Emma Colvert, Emily Simonoff, Simone J. Capp, Angelica Ronald, Patrick Bolton, Francesca Happé

**Affiliations:** 1 Social, Genetic, and Developmental Psychiatry Centre, Institute of Psychiatry, Psychology, and Neuroscience, King’s College London, PO80, Denmark Hill, London, SE5 8AF UK; 2 Department of Child and Adolescent Psychiatry, Institute of Psychiatry, Psychology, and Neuroscience, King’s College London, Denmark Hill, London, SE5 8AF UK; 3 Department of Psychological Sciences, Birkbeck, University of London, Malet Street, London, WC1E 7HX UK

**Keywords:** Autism spectrum disorders, Mental health, Longitudinal research, Adolescents

## Abstract

There is increasing concern regarding additional psychiatric problems that co-occur with Autism Spectrum Disorder (ASD), as reflected in recent changes to diagnostic schemes. However, there remains little research with population-based samples across childhood. We report on additional problems, as measured by the Strengths and Difficulties Questionnaire, in a population-based sample of 135 twins with ASD, 55 non-ASD co-twins, and 144 comparison twins low in ASD traits. Frequencies, associated demographic factors, and changes in mental health difficulties from age 4 to 13 years are presented. Our data confirm the high rates of additional difficulties reported in previous studies, and suggest that the profile, associated risk factors and longitudinal course of additional difficulties in ASD may differ from those in typically-developing populations.

**Supplementary Information:**

The online version contains supplementary material available at 10.1007/s10803-021-05006-8.

Autism Spectrum Disorders (ASD) are lifelong neurodevelopmental conditions characterised by social and communicative difficulties and restricted, repetitive behaviours and interests. Approximately 1% of children and adults have an ASD diagnosis. In addition to the defining features of ASD, it is increasingly clear from recent research that autistic individuals commonly show additional psychiatric conditions. Since additional disorders may add to the burden for these individuals and their carers and may be amenable to treatment, recognition and diagnosis are important. This is reflected in changes to the latest edition of the APA’s Diagnostic and Statistical Manual (DSM-5; APA, 2013), which allows multiple diagnoses alongside ASD (e.g., ADHD and Anxiety Disorder) for the first time.

A large number of studies have reported evidence of raised rates of additional psychiatric difficulties in ASD. However, most have focused on clinic samples (e.g., Brereton et al., 2006; Ghaziuddin & Greden, 1998; Joshi et al., 2010, 2013; Kim et al., 2000; Larsen & Mourisden, 1997; Mukaddes et al., 2010; Sukhodolsky et al., 2008), which are known to be biased towards more complex or severely affected cases (Caron & Rutter, 1991). From these previous studies some consistent findings have emerged: ADHD and hyperactive type behaviour have commonly been found to co-exist with ASD (Goldstein & Schwebach, 2004; Kim et al., 2000; Leyfer et al., 2006; Rosenberg et al., 2011; Simonoff et al., 2008), as have Tourettes (Ehlers & Gillberg, 1993; Kerbeshian & Burd, 1996; Nass & Gutman, 1997), depression (Cederlund & Gillberg, 2004; Green et al., 2000; Kim et al., 2000; Leyfer et al., 2006; Rosenberg et al., 2011; Shtayermman, 2007), anxiety disorder (Joshi et al., 2013; Mukaddes et al., 2010; Rosenberg et al., 2011; Shtayermman, 2007; Simonoff et al., 2008; Sukhodolsky et al., 2008) and OCD (Cath et al., 2008; Green et al., 2000; Leyfer et al., 2006; Tantum, 1991). Other somewhat less common comorbid conditions are phobias (Green et al., 2000; Leyfer et al., 2006; Muris et al., 1998), tics (Ehlers & Gillberg, 1993), eating disorders (Wentz et al., 2005) and schizophrenia (Konstantereas & Hewitt, 2001; Larsen & Mouridsen, 1997; Tantum, 1991). In addition, many individuals with ASD have been found to have more than one additional psychiatric problem (e.g., Lai et al., 2019; Leyfer et al., 2006).

In order to get a more accurate estimate of the rates and patterns of additional difficulties across the full spectrum of ASD, data from population-based studies are vital. To date, very few population-representative studies have been conducted. Lundström et al. (2011) examined two large nationally representative Swedish twin cohorts (one adult and one child) to examine links between autistic like traits (as assessed using DSM-IV based algorithms) and co-occurring mental health difficulties (ADHD, conduct problems and anxiety/depression, as assessed using the Autism-Tics, ADHD and other Comorbidities inventory). The study found links between the levels of autism like traits and the occurrence of mental health problems, with even slight increases in traits being a risk factor for emotional and behavioural difficulties. Totsika et al. (2011), looked cross-sectionally at levels of emotional and behavioural problems for five- to 16-year-olds in a population-representative study and found elevated levels for those with ASD, regardless of level of intellectual functioning, when compared to a comparison group. A study that incorporated a whole population approach was carried out by Rydzewska et al. (2019), whereby Scottish census data for 2011 was investigated to assess the odds ratios for autism, in those aged 0–24 years, predicting sensory, physical and intellectual disabilities and mental health comorbidities. In terms of mental health difficulties these were found to be substantially (15 times) more common in the population with autism when compared to the general population.

One notable population-based study using standardised assessments was carried out by Simonoff et al. (2008, 2013) who found that 70% of ten- to 14-year-olds with ASD met criteria for at least one disorder, with 41% having two or more problems in addition to ASD. The most common disorders to co-occur with ASD were social anxiety, hyperactivity and oppositional defiant disorder. Simonoff et al. (2008, 2013) also examined links to risk factors (e.g., SES, IQ) commonly found to be relevant in (non-ASD) childhood psychiatric research, but found no clear links to additional problems in ASD, perhaps indicating differences in the origins or nature of these disorders in ASD versus those in the general population.

The current study reports rates and patterns of additional problems in a population-based sample of twins with ASD, over a nine-year time span. The research is part of the Social Relationships Study (SR Study) which is one of the largest ever population-based twin studies of ASD encompassing the full range of the autism spectrum. The study population had a mean age of 13 years at the time of the SR Study home visits/questionnaires, which can be a key time for the emergence of psychiatric difficulties in the general population (Bohman & Sigvardsson, 1980; Brodzinsky, 1993; Verhulst & Versluis-Den Bieman, 1995).

In addition to the ASD sample, the SR Study includes the non-ASD co-twins in the ASD families, and a matched comparison sample selected to be low in ASD traits. Co-twins of children with ASD share family-wide factors (e.g., SES) and therefore act as a valuable comparison sample whose difficulties are reported by the same parent group. There is also considerable interest in the Broader Autism Phenotype and mental health, and it is valuable to examine whether rates of additional problems are elevated in the non-ASD co-twins of autistic children.

The fact that the SR Study includes a twin population allows for an investigation of the possible role of genetics in comorbidity. It is well established that there is a strong genetic component to ASD (e.g., Bailey et al., 1995; Colvert et al., 2015; Rutter, 2000). However, it is unclear whether there is a similar and/or overlapping genetic influence on psychiatric conditions that co-occur with ASD (see Hallett et al., 2013). The literature on autistic traits has been valuable for demonstrating degree of shared genetic and environmental influences with other psychopathology traits such as ADHD traits and anxiety-related behaviours both in the same twin population from which the SR Study derives and other population-based twin studies (Hallett et al., 2010; Lundström et al., 2011; Polderman et al., 2014).While the present paper presents phenotypic data, twin analyses based on an overlapping data set are presented elsewhere (Tick et al., 2016).

The SR Study is nested within the larger Twins Early Development Study (TEDS) and benefits from the wealth of longitudinal data collected for twins in TEDS from age 2 onwards. The current study makes use of these previous data by examining the longitudinal course of the domains of possible comorbidity, measured by the parent-rated Strengths and Difficulties Questionnaire (SDQ), to investigate changes in additional difficulties over time. The SDQ is a widely used measure of psychopathology both clinically and in research settings. It has been shown to have good reliability and validity for both typically developing populations (Goodman, 2001; Muris et al., 2003; Lundh et al., 2008; Yao et al., 2009) and within autistic samples (Findon et al., 2016). Data for a subset of participants with SDQ scores from ages 4, 7 and the SR Study time point (c. 13 years) are examined. To date there have been few studies documenting longitudinal analyses of comorbidities in ASD (e.g., Gray et al., 2012; Libove et al., 2017; Saito et al., 2017). Simonoff et al. (2013) compared rates of comorbid difficulties at ages 12 and 16 years in their population-based ASD cohort, and found that difficulties were persistent across the two time points and, as in their previous research, not linked to risk factors identified in typically developing samples. The current study includes data from three time points to extend the work of Simonoff et al. (2013) and to provide the first attempt to replicate their findings in an independent, population-based sample.

In summary, the existence of co-occurring or comorbid conditions in ASD has been shown in numerous clinic-based studies and in autistic trait-based research, but more population-based studies of ASD are needed. The current study aims to establish, for the first time, the rates and patterns of co-occurring difficulties in a large population-based twin sample covering the full range of the autism spectrum, to examine possible risk factors and the longitudinal course of difficulties shown in ASD and comparison twins.

## Methods

### Participants

The SR Study sample was initially drawn from the TEDS population (see Haworth et al., 2013 for details) and aimed to include all families in which one or both twins were suspected or confirmed to have ASD. Full details of the sample ascertainment can be found in Colvert et al. (2015).

In order to categorise the sample, the gold-standard diagnostic tools of the Autism Diagnostic Observation Schedule (ADOS-G Lord et al., 1989) and Autism Diagnostic Interview (ADI-R Rutter et al., 2003) were used: in total ADOS-G assessments were conducted for 254 children and ADI-R interviews were carried out for 241. The use of different diagnostic tools was an advantage of the study as it allowed comparison of parent and observer rated measures of autistic symptoms. However, the differences between these tools meant that they did not converge on the same diagnosis for all cases; there were 89 cases (37%) where there was disagreement between the two measures. All cases with diagnostic disagreement were referred to a team of psychiatrists who reviewed the available data sources, including previous information gathered by TEDS about diagnoses, and reached a best estimate diagnosis according to DSM-IV criteria.

A comparison group was also included in the SR Study, consisting of 79 families from the main TEDS population who scored below 12 on the Childhood Autism Spectrum Test (CAST; Scott et al., 2002) at age 8 years and who lived in the South East of England. The comparison group were matched to the group of all suspected ASD cases (i.e. all families who took part in the study where at least one twin had ASD, N = 190 twins) in terms of sex (ASD sample 72% male; comparison sample 69% male), zygosity (ASD sample 22% MZ, 37% DZ same sex, 41% DZ opposite sex; comparison sample 33% MZ, 32% DZ same sex, 33% DZ opposite sex), and SES (ASD sample mean SES 0.2; comparison sample mean SES 0.1, see below for a description of how SES was calculated). There was a small but significant difference in terms of age (ASD sample mean age 13.5 years; comparison mean age 12.7 years, t = 6.48; df236; p < 0.001), hence age is included as a factor throughout the current study. A sub-sample of the comparison group were selected (N = 82 individuals) who were more closely matched to the final ASD sample in terms of sex ratio (85% male) and age (mean age 160 months) and for key analyses no differences were found when using this sub-sample or the full comparison group. The comparison group received the same measures as the ASD and co-twin sample minus the ADOS-G and ADI-R diagnostic assessments (Table 1).

**Table 1:** Participant details

Group	Age M (SD)	Sex N (%)	Zygosity N (%)	SES M (SD)	IQ M (SD)
ASD N = 135	13 years 5 months (8.69 months)	18 Female (13%) 117 Male (87%)	39 MZ (29%) 50 DZ same sex (37%) 46 DZ opp sex (34%)	0.19 (0.78)	92 (24.90)
Co-twin N = 55	13 years 6 months (8.36 months)	35 Female (64%) 20 Male (36%)	3 MZ (6%) 20 DZ same sex (36%) 32 DZ opp sex (58%)	0.31 (0.78)	105 (13.17)
Comparison N = 144	12 years 9 months (13.15 months)	45 Female (31%) 99 Male (69%)	48 MZ (33%) 46 DZ same sex (32%) 50 DZ opp sex (35%)	0.15 (0.62)	103 (14.82)

### Measures

#### *Strengths and Difficulties Questionnaire (SDQ) (*Goodman, 1997*)*

Emotional and behavioral difficulties were measured using the 25 item parent completed SDQ (Goodman, 1997). For each participant the SDQ questionnaire was completed by the same parent who completed the ADI-R and other interviews for the SR Study, in the vast majority of cases this was the mother of the children (97%).

Each item on the SDQ is scored 0 for does not apply, 1 for applies somewhat or 2 for certainly applies. Composite scores were calculated for five separate domains: emotional difficulties, conduct problems, hyperactivity, peer problems and pro-social behaviour. Peer and pro-social domains are clearly related to the core symptoms of ASD and cannot be considered as additional psychiatric difficulties in this group. However, since ASD-like traits in co-twins are of interest, information about all five domains are reported below, except where counts are made of additional psychiatric difficulties.

Both domain scores and cut-off scores are included in the analyses, with cut-offs based on the new (somewhat higher) published population-based norms outlined in the 2016 scoring guidelines for the SDQ (see supplementary materials Appendix 1 for details of the cut-offs used for each domain in the current study).

#### *Autism Diagnostic Interview Revised (ADI-R) (*Rutter et al., 2003*)*

The ADI-R is a gold-standard diagnostic tool for the assessment of ASD. It comprises a semi-structured interview of 93 items, carried out by a trained investigator over the course of 2–3 h. It is designed to give a lifetime differential diagnosis of ASD, taking into account current behaviours and characteristics and the presentation of autistic symptoms earlier in childhood. Researchers involved in the current study were trained extensively in ADI-R administration methods and completed regular inter-rater reliability meetings to ensure the accuracy of both administration and scoring. The analyses included in the current study use the total ADI-R score.

#### *Autism Diagnostic Observation Schedule (ADOS-G) (*Lord et al., 1989*)*

The ADOS-G is a gold-standard diagnostic observational play and activity-based assessment, with different modules for differing ability and language levels. Researchers carrying out the current study were all trained extensively in the administration and scoring of the ADOS-G and completed regular inter-rater reliability meetings to ensure accuracy of administration and scoring throughout the study. The current study used the most recent algorithms for ADOS-G scoring at the time (provided by C. Lord, equivalent to current ADOS-2 algorithms) and these yielded scores for communication, social interaction and stereotyped behaviours and restricted interests. In the current analyses the calibrated severity score (CSS) is used to allow comparison across all ADOS-G modules used.

#### Intellectual Ability

Intellectual ability was assessed using the Wechsler Abbreviated Scale of Intelligence (WASI; Wechsler, 1999) to obtain an estimated score for full scale IQ. Fourteen nonverbal participants instead completed the Raven's Coloured Progressive Matrices (Raven et al., 1998) and the British Picture Vocabulary Scales-Revised (BPVS) (Dunn et al., 1997) to obtain an estimated score for verbal and performance IQ. To include the low IQ individuals in the subsequent analyses, a full scale IQ equivalent score was calculated for them using the age equivalent scores of BPVS and Ravens using the formula: (age equivalent/chronological age) multiplied by 100. For those with extremely low raw scores, the age equivalent of 24 months was entered as the floor level for measurement on the BPVS. Similarly, the age equivalent of 47 months was entered as a floor level for measurement for the Ravens. When reporting IQ in analyses this is a composite measure using WASI where this was available and the combined full scale IQ (BPVS as VIQ and Ravens as NVIQ) for those without WASI scores.

#### *Childhood Autism Spectrum Test (CAST) (*Scott et al., 2002*)*

The CAST was sent to all participants in the current study and it forms part of the participant sub-group selection process (see Colvert et al., 2015 for details). The CAST is a 31 item parent-completed questionnaire requiring yes/no responses. The questionnaire yields a total score which is then compared to a population-based norm cut-off of 15. In the current analyses, CAST total scores are used to estimate degree of ASD-like traits in the comparison sample, since this group did not complete the ADI-R or ADOS-G. Note that the comparison group was originally selected for low ASD traits on the CAST (< 12 points) at age 8.

#### Socio-Economic Status (SES)

The family SES measure included in analyses is frequently used in TEDS (e.g., Petrill et al., 2004, which details the creation of this variable); a composite of maternal age at birth of eldest child, parental highest education level, and parental occupation when the twins were aged 2 years (the time when demographic information was first collected in TEDS).

## Results

Throughout the results section the participants are treated as singletons (i.e. each member of the twin pairs is included in analyses), to maximise the power to compare the ASD, co-twin and comparison samples. In order to account for non-independence of data points, analyses were re-run taking one twin from each family at random; the pattern of results and significance was not altered. Due to unequal variances and non-normal distribution of some variables, non-parametric statistics were employed (skewness and kurtosis values for the SDQ distributions are included in the supplementary materials, Appendix 2). Where possible, effect sizes are reported as appropriate for the various statistical tests, following the guidelines of Tomczak and Tomczak (2014). Bonferroni corrections for multiple comparisons are included for all group comparisons and full results of statistical analyses can be found in the supplementary materials.

### Descriptive Information on SDQ-Rated Domains

The mean scores for the three sample groups (ASD, co-twins and comparison) for each domain of the SDQ (emotional, conduct, hyperactivity, peer and pro-social) are shown in Table 2. As can be seen, for all domains the ASD group show significantly worse scores than the other two groups (note that the pro-social scale is reversed so lower scores show more severe problems). The co-twins and comparison groups did not differ significantly in any of the domains. Results were examined controlling for IQ and all group differences were found to remain (all p’s < 0.05 using Quade’s rank analysis of covariance).

**Table 2:** Mean (SD) SDQ domain scores by group

Group	Domains of SDQ				
	Emotional	Conduct	Hyperactivity	Peer	Pro-social
ASD N = 135	3.95 (2.55)	2.21 (2.23)	5.63 (2.87)	4.91 (2.55)	5.88 (2.95)
Co-twin N = 55	1.76 (1.64)	1.09 (1.36)	2.62 (2.45)	0.89 (1.29)	8.62 (1.64)
Comparison N = 144	1.53 (1.75)	1.31 (1.28)	3.08 (2.19)	0.82 (1.36)	8.51 (1.48)
Kruskal–Wallis statistic	χ 2 (2) = 76.91, p < .001, ƞ2 = 0.23	χ 2 (2) = 16.83, p < .001, ƞ2 = 0.05	χ 2 (2) = 68.09, p < .001, ƞ2 = 0.20	χ 2 (2) = 169.56, p < .001, ƞ2 = 0.51	χ 2 (2) = 73.28, p < .001, ƞ2 = 0.22

For all domains Bonferroni corrected post hoc Dunn analyses showed that the ASD group differed significantly from both the co-twin and the comparison groups (all p < .01), no differences were seen between the co-twins and the comparison groups (all p > .49) (see supplementary materials Appendix 3 for full details of post hoc results)

In addition to the domain score data, the SDQ also affords cut-off scores to indicate those with the most extreme difficulties. For the current study, only one cut-off category was used, which was a combination of those rated ‘high’ and ‘very high’ on the latest published norms; this was done as the high cases showed the same profile of results as the very high group and were few in number. The amalgamation of the groups, which is broadly equivalent to taking the “abnormal” category from the older three-fold categorical system for the SDQ, made no difference to the pattern of significant results obtained. Table 3 shows the frequencies and percentages for those above cut-off in each domain of the SDQ for each group. As can be seen, the ASD group showed higher rates of above cut-off difficulties than the co-twin and the comparison groups, in all domains. The chi-square analyses show that the results for all domains were significant and additional post hoc chi-square analyses showed that the higher ASD rates are driving this result, with the co-twins and comparison sample showing broadly similar rates of difficulties.

**Table 3:** Numbers (%) participants scoring above cut-off on SDQ domains

Group	Domains of SDQ				
	Emotional	Conduct	Hyperactivity	Peer Problems	Pro-social
ASD N = 135	52 (39%)	32 (24%)	44 (33%)	99 (73%)	72 (53%)
Co-twin N = 55	3 (6%)	4 (7%)	3 (6%)	3 (6%)	7 (13%)
Comparison N = 144	12 (8%)	9 (6%)	7 (5%)	6 (4%)	15 (10%)
	χ 2 (2, N = 334) = 48.35, p < .001, V = .38	χ 2 (2, N = 334) = 20.38, p < .001, V = .25	χ 2 (2, N = 334) = 45.11, p < .001, V = .37	χ 2 (2, N = 334) = 174.10, p < .001, V = .72	χ 2 (2, N = 334) = 71.20, p < .001, V = .46

For all domains post hoc chi-square analyses showed that the ASD group differed significantly from both the co-twin and the comparison groups (all χ 2 s above 6.86, all p < .01), no differences were seen between the co-twins and the comparison groups (all p > .48) (see supplementary materials Appendix 3 for full details of post hoc results)

### Multiple Problems

**Fig. 1:**
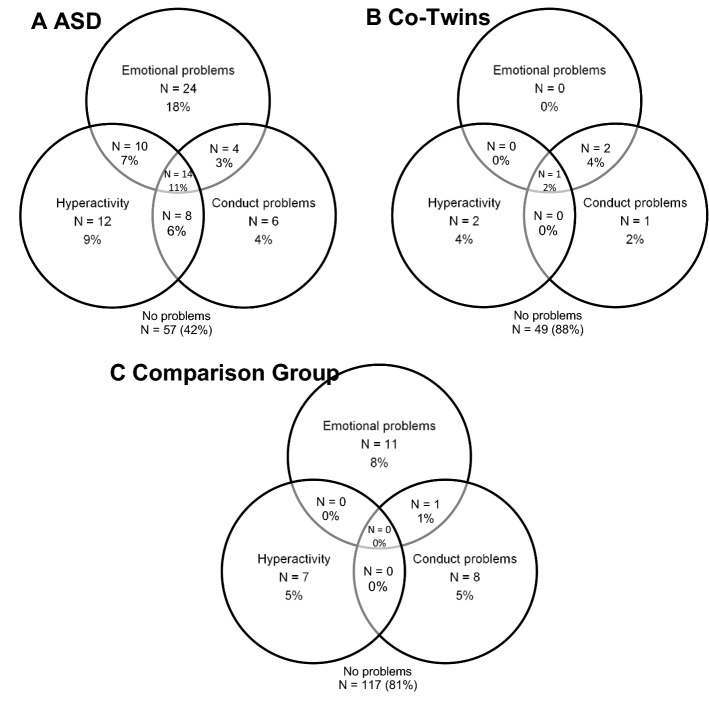
Venn diagrams for multiple problems (passing cut-offs on the SDQ)

We examined the frequencies of multiple problems (i.e. multiple scores above cut-off) in each sample group. These analyses were limited to the emotional, conduct and hyperactivity domains, since peer and pro-social problems are not independent of the ASD profile and might inflate the count. As can be seen in Table 4, the majority of the comparison sample and co-twins show either zero or one problem only, however, many of the ASD group show multiple problems. Overall, 58% of the ASD sample (N = 78) showed at least one problem and taking the sample as a whole 27% (N = 36) had multiple difficulties. In contrast, 11% (N = 6) of the co-twins showed at least one problem with only 6% (N = 3) having more than one, and 19% (N = 27) of the comparison sample showed at least one problem area, with just 1% (N = 1) having more than one. Figure 1 shows the differing compositions of multiple problems seen for the sample groups.

**Table 4:** Frequencies (N (%)) and descriptives (m (SD)) for number of domains of the SDQ with above cut-off ratings

	Number of domains of the SDQ with above cut-off ratings			
Group	0	1	2	3
ASD	N = 57 (42%)	N = 42 (31%)	N = 22 (16%)	N = 14 (11%)
Mean age	161 (7.51)	161 (10.74)	160 (8.62)	165 (5.97)
Mean IQ	97 (20.29)	89 (29.38)	77 (25.29)	98 (19.33)
Mean ADI-R	34.57 (16.99)	40.22 (14.27)	44.27 (11.53)	38.79 (14.77)
Mean ADOS CSS	5.96 (2.60)	5.48 (2.84)	6.26 (2.38)	7.38 (2.53)
Co-twin	N = 49 (89%)	N = 3 (5%)	N = 2 (4%)	N = 1 (2%)
Mean age	162 (8.65)	161 (6.08)	169 (1.41)	158
Mean IQ	107 (12.34)	102 (22.19)	88 (7.07)	89
Mean ADI-R	4.51 (3.80)	6.67 (5.03)	8.00	15.00
Mean ADOS CSS	1.34 (0.89)	1.67 (1.15)	1.50 (0.71)	1.00
Comparison	N = 117 (81%)	N = 26 (18%)	N = 1 (1%)	N = 0 (0%)
Mean age	153 (13.16)	152 (12.47)	180	
Mean IQ	103 (15.72)	102 (10.10)	90	
Mean CAST	4.60 (2.74)	4.70 (2.02)	7.00

### Possible Risk Factors for SDQ-Rated Problems

The relationship between the SDQ domain scores and cut-off ratings and the following six risk factors was examined; age, full scale IQ, SES, sex, zygosity and a measure of ASD severity (for the ASD and co-twin groups this was ADI-R total algorithm score and ADOS CSS, for the comparison group this was CAST score as they did not complete the ADI-R or ADOS-G). The risk factor analyses were carried out both dimensionally (using Spearman correlations with the domain scores – see supplementary materials Appendix 3 for full correlation tables) and dichotomously (using logistic regression to assess what predicted a rating above versus below cut-off).

For the domain scores for the ASD group ADI-R, ADOS-G and IQ showed links to hyperactivity (ADI-R: rs (133) = 0.34, p < 0.001; ADOS-CSS: rs (128) = 0.19, p = 0.036; IQ: rs (121) = − 0.22, p = 0.017), peer problems (ADI-R: rs (133) = 0.44, p < 0.001; ADOS-CSS: rs (128) = 0.32, p < 0.001; IQ: rs (121) = − 0.24, p = 0.008) and pro-social (ADI-R: rs (133) = − 0.43, p < 0.001; IQ: rs (121) = 0.18, p = 0.047), with ADOS-CSS almost reaching significance for pro-social (ADOS-CSS: rs (128) = − 0.17, p = 0.057). In terms of the cut-off scores, ASD severity only (as measured by the ADI-R) played a role in predicting hyperactivity and peer problems (hyperactivity: χ2 (7) = 18.08, p = 0.012, B = 0.06, SE = 0.03, exp(B) = 1.06, p = 0.05; peer problems: χ 2 (7) = 18.02, p = 0.012, B = 0.09, SE = 0.03, exp(B) = 1.10, p = 0.002).

For the co-twin group’s domain scores, ADI-R ratings were related to two areas (emotional: rs (54) = 0.32, p = 0.020; peer: rs (54) = 0.33, p = 0.015). SES showed links to conduct and hyperactivity (conduct: rs (43) =− 0.55, p < 0.001; hyperactivity: rs (43) = − 0.49, p = 0.001). IQ showed one significant association with hyperactivity (rs (53) = − 0.29, p = 0.033). In terms of the regression analyses, no significant predictors of passing cut-off were found in the co-twin group.

For the comparison sample there was a varied range of relationships shown for the domain scores, with CAST showing links to all SDQ domains except emotional (conduct: rs (140) = 0.35, p < 0.001; hyperactivity: rs (140) = 0.32, p < 0.001; peer problems: rs (140) = 0.18, p = 0.039; pro-social: rs (140) = − 0.23, p = 0.005). SES was linked to conduct and hyperactivity (conduct: rs (142) = − 0.17, p = 0.044; hyperactivity: rs (142) = − 0.24, p = 0.005) and IQ showed links to three areas for this group (conduct: rs (143) = − 0.19, p = 0.021; hyperactivity: rs (143) = − 0.35, p < 0.001; peer problems: rs (143) = − 0.21, p = 0.013). Finally, age showed a relationship with both conduct and peer problems (conduct: rs (144) = 0.22, p = 0.007; peer: rs (144) = 0.19, p = 0.020). The comparison group showed two significant results for the cut-off regression analyses, with CAST and SES significant predictors of passing cut-off for pro-social ratings (CAST: χ 2 (6) = 16.05, p = 0.013, B = 0.25, SE = 0.12, exp(B) = 1.3, p = 0.036; SES: χ 2 (6) = 16.05, p = 0.013, B = − 1.61, SE = 0.76, exp(B) = 0.20, p = 0.033).

Sex and zygosity were also examined in terms of links to the domains of the SDQ. Zygosity was found to play a significant role for hyperactivity in the ASD group only, with the frequencies indicating that the DZ group were more likely to be rated above cut-off than the MZ group (χ 2 (7) = 18.08, p = 0.012, B = − 2.15, SE = 1.09, exp(B) = 0.12, p = 0.049). Sex was found to have an impact for two areas; hyperactivity for the co-twins (U = 493, p = 0.011, r = 0.34, with males showing higher scores) and pro-social for the comparison group (U = 1654, p = 0.011, r = 0.21, with females showing higher scores and therefore fewer problems). In order to examine possible sex effects further, given the unequal sex ratios across groups, within-sex patterns of results were examined. Taking females and males separately, both sexes showed the same pattern of SDQ domain scores and SDQ cut-off results, with the same significant group differences occurring, indicating that sex was not a confounding factor.

### Longitudinal Findings

Due to the longitudinal nature of TEDS, SDQ data were elicited from the SR Study sample parents at various time points before the SR Study home visits at mean age 13 years and 2 months (age range 12–14 years). The data gained at ages 4 and 7 years were examined together with those from age 13 years to elucidate the longitudinal pattern of comorbid difficulties. The longitudinal data for the comparison and co-twin samples were included for contrast. Only those participants with data from all three time points are included in this section of analyses. In order to examine whether there were any systematic differences between those with and without data available for ages 4 and 7 years, the two samples were compared in terms of SDQ domain scores, SES and IQ. The ASD sample without age 4 and 7 data was significantly lower in IQ (U = 2338, p = 0.004, r = 0.26) and showed a higher level of peer problems on the SDQ (U = 1454, p = 0.001, r = 0.30), compared to the ASD sample with data from all three time points. The lower IQ finding reflects the fact that the SR Study sought to include the full range of the autism spectrum and therefore included families who may not have taken part in earlier TEDS assessments. The co-twin and comparison groups showed no differences for those with complete longitudinal data versus those with data only available from the SR Study time point.

#### Longitudinal Domain Scores

Table 5 shows the mean SDQ domain scores for the three time points for each participant group. The table shows that at each time point the ASD group exhibited higher scores than the other two groups for all domains. Kruskal–Wallis statistics revealed that at ages 4 and 7 years the pattern was the same as the most recent SR Study data collection (Age 4: all χ 2 > 7.30, all ps < 0.05, all ƞ2 s > 0.01, range 0.02–0.25; Age 7: all χ 2 > 22.33, all ps < 0.001, all ƞ2 s > 0.08, range 0.09–0.37, see supplementary materials Appendix 3 for full details of results); all domains showed significant group differences, with post hoc Bonferroni corrected Dunn tests showing higher scores in the ASD group (Age 4: all ps < 0.05; Age 7: all ps < 0.01), whilst the co-twins and comparison group did not differ significantly.

**Table 5:** Mean scores and frequency above cut-off for each SDQ domain over the three time points

Group	Domains of SDQ				
	Emotional	Conduct	Hyperactivity	Peer problems	Pro-social
ASD N = 78					
Age 4	2.21 (2.09) 18%	2.91 (1.70) 33%	5.24 (3.02) 26%	3.68 (2.30) 54%	6.42 (2.51) 47%
Age 7	3.86 (2.52) 41%	3.04 (2.16) 42%	6.42 (2.74) 45%	3.98 (2.79) 49%	6.50 (2.48) 44%
SR study (mean age 13yrs 2mths)	3.86 (2.50) 40%	2.33 (2.42) 26%	5.51 (2.93) 33%	4.27 (2.42) 65%	6.23 (2.83) 49%
Co-twin N = 35					
Age 4	1.43 (1.56) 6%	1.94 (1.45) 17%	2.94 (2.13) 3%	1.31 (1.57) 9%	7.86 (1.93) 26%
Age 7	2.70 (1.98) 17%	1.74 (1.52) 11%	3.09 (2.56) 6%	0.86 (1.14) 3%	8.17 (1.84) 17%
SR study (mean age 13yrs 2mths)	1.57 (1.56) 3%	1.09 (1.36) 6%	2.51 (2.50) 6%	0.66 (1.16) 6%	8.51 (1.79) 14%
Comparison N = 111					
Age 4	1.34 (1.43) 4%	2.07 (1.44) 15%	3.69 (2.30) 4%	1.32 (1.45) 9%	7.57 (1.87) 24%
Age 7	2.03 (1.99) 14%	1.65 (1.41) 11%	3.57 (2.55) 9%	0.84 (1.44) 7%	8.23 (1.74) 20%
SR study (mean age 13yrs 2mths)	1.58 (1.75) 8%	1.23 (1.20) 4%	3.09 (2.22) 5%	0.90 (1.46) 5%	8.59 (1.39) 8%

Changes in domain scores within group across time points were also examined, with repeated measures Friedman analyses. The ASD group showed three significant time differences (emotional: χ2 (2) = 29.79, p < 0.001, w = 0.19; conduct: χ2 (2) = 15.53, p =  < 0.001, w = 0.10; hyperactivity: χ2 (2) = 12.48, p = 0.002, w = 0.08), Table 5 details this fluctuation, showing a relative peak for difficulties in all areas at age 7, although pair-wise comparisons indicated that scores at age 7 were not significantly higher than those at ages 4 and the SR Study time point (c. 13 years) across all three domains. The co-twins showed only two significant time differences (emotional: χ2 (2) = 20.20, p =  < 0.001, w = 0.29; conduct: χ2 (2) = 10.04, p = 0.007, w = 0.14); Table 5 shows that for emotional problems there was a peak at age 7 (pair-wise comparisons showed that age 7 scores were significantly higher than those for age 4 and the SR Study time point (c. 13 years), all p’s < 0.01) and that conduct problems decreased over time for co-twins. The comparison group showed significant differences over time, with the pattern broadly being one of reductions in difficulties for all domains(although emotional difficulties showed a slightly different pattern with the highest levels seen at the age 7 time point) (emotional: χ2 (2) = 10.84, p = 0.004, w = 0.05 (age 7 > age 4, p = 0.016); conduct: χ2 (2) = 22.01, p < 0.001, w = 0.10 (age 4 > age7, p < 0.001); hyperactivity: χ2 (2) = 9.17, p = 0.010, w = 0.04 (age 4 > SR Study(c. 13 years), p = 0.036); peer problems: χ2 (2) = 12.47, p = 0.002, w = 0.06 (age 4 > age7, p = 0.024); pro-social: χ2 (2) = 22.91, p < 0.001, w = 0.10 (age4 > age7, p = 0.024; age4 > SR Study(c. 13 years), p < 0.001).

#### Longitudinal Cut-off Scores

Table 5 shows the frequencies of above cut-off ratings for each group across the three time points. As can be seen, there was a good deal of fluctuation in the rates of above cut-off difficulties for all the groups, with the comparison and co-twin groups showing higher rates at earlier time points, whereas the ASD sample appears more stable over time. The group differences for rates of above cut-off scores were examined for each of the two earlier time points and were found to be significant for all domains (Age 4: all χ2 s above 9.24, all ps < 0.01; Age 7: all χ2 s above 15.22, all ps < 0.001, see supplementary materials Appendix 3 for full details of results). As with the later SR Study time point the group differences at ages 4 and 7 are driven by the higher rates for the ASD sample.

Next the pattern of ratings above cut-off over time was analysed using Cochran’s Q tests. The ASD sample showed significant differences for four domains (emotional: χ2 (2) = 14.62, p = 0.001; conduct: χ2 (2) = 6.87, p = 0.032; hyperactivity: χ2 (2) = 9.00, p = 0.011; peer problems: χ 2 (2) = 7.02, p = 0.030); Table 5 and post hoc analyses show that for emotional problems there were significant increases between age 4 rates above cut-off and those at age 7 (p = 0.002) and the SR Study time point (c. 13 years) (p = 0.004), with the latter two time points not differing significantly. For the conduct ratings there was a slight (non-significant) increase between age 4 and 7 then a significant decrease towards the SR Study time point (c. 13 years) (p = 0.027). For hyperactivity there was a significant increase between ages 4 and 7 (p = 0.009). For peer problems there was a significant increase in the ratings above cut-off between ages 7 and the SR Study time point (c. 13 years) (p = 0.027). The co-twins showed no significant differences over the three time points. The comparison group showed significant differences for three domains (emotional: χ2 (2) = 8.27, p = 0.016; conduct: χ2 (2) = 10.32, p = 0.006; pro-social: χ2 (2) = 18.50, p < 0.001). Table 5 and post hoc analyses show that for conduct and pro-social problems there was a decrease in ratings above cut-off from age 4 to both the age 7 and SR Study (c. 13 years) time points (all post hoc p’s < 0.01), while emotional problems showed a spike at age 7.

#### Longitudinal Pattern of Difficulties

**Fig. 2:**
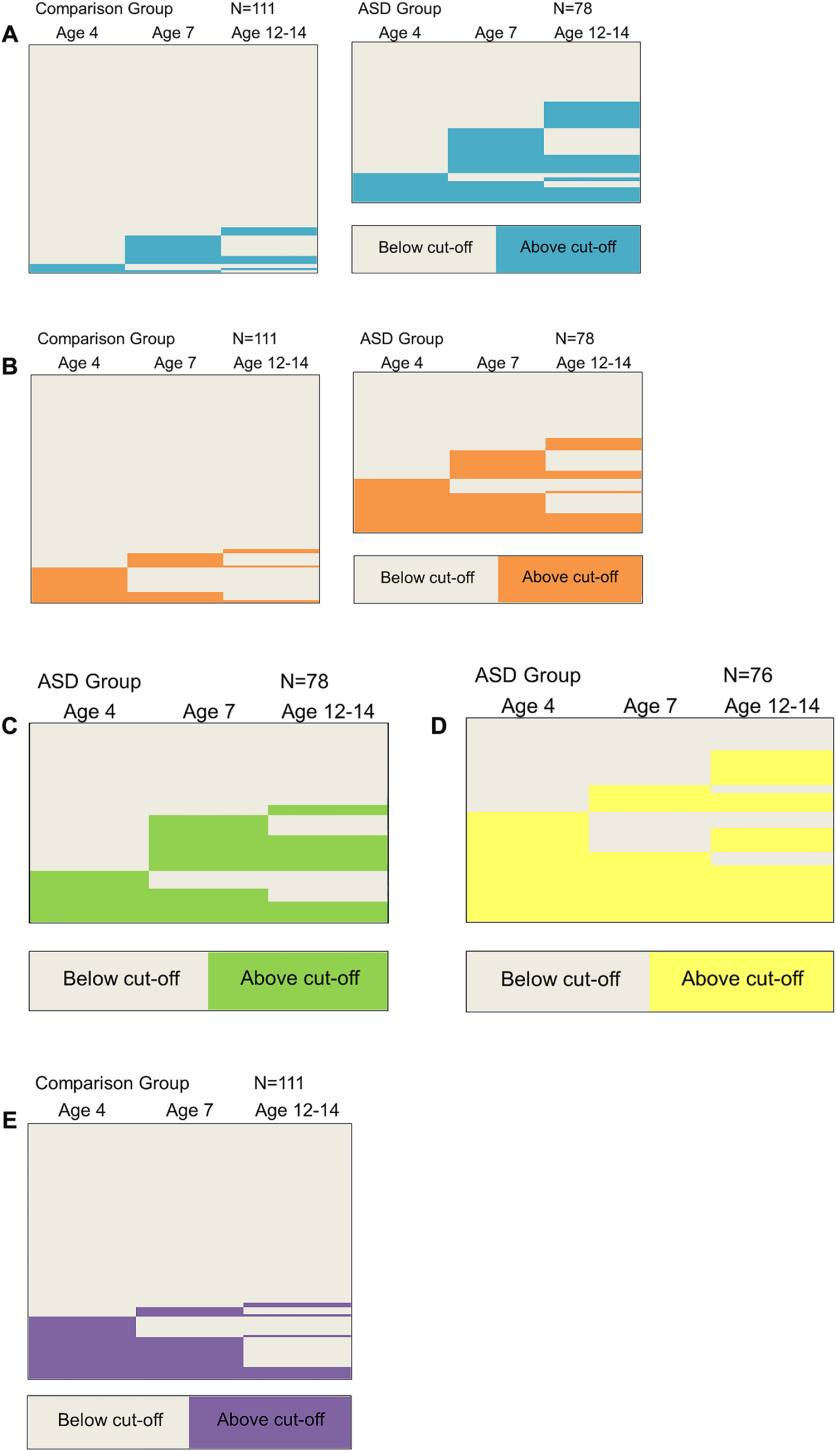
Longitudinal patterns of above cut-off SDQ problem scores (note each line represents one participant). **a** Emotional domain. **b** Conduct domain. **c** Hyperactivity domain. **d** Peer domain. **e** Pro-social domain

In order to examine the changes over time in more detail at the individual level, the domains showing significant differences across the three time points were examined to ascertain the composition of the differences, i.e. whether it was caused by onset, offset or persistence of ratings above cut-off, as shown in Fig. 2 (in which each line represents a single participant). As the co-twins did not show any significant differences over time their results are not included in this section. Results reported in this section reflect observed trends. For the ASD sample the emotional difficulties domain showed most onset at ages 7 and the SR Study time point (c. 13 years), and most persistence from age 7 to the SR Study time point (c. 13 years); there were only seven (14% of those showing problems in the domain at any time) persistors across all three time points. Conduct problems showed slightly more stability over time, with nine persistors, accounting for 20% of all those showing any problems in this domain over the three time points, and similar levels of onset for all three time points. Hyperactivity showed most onset at age 7 and a 17% persistence rate over the three time points. Peer problems revealed the largest amount of persistence with 32% of those showing problems at any point passing cut-off at all three time points. The comparison group showed significant differences for emotional problems and, like the ASD group, most onset occurred at the later time points of age 7 or the SR Study time point (c. 13 years). Unlike the ASD group, none of the comparison group showed persistence across the three time points for emotional problems. Conduct difficulties also showed a very different pattern to that in the ASD sample (the pattern was significantly different when comparing the two groups for rates of onset, offset and persistence: χ2 (7, N = 189) = 36.12, p < 0.001, V = 0.44). Most comparison children with any conduct disorder showed onset at age 4 (65% of all those showing conduct difficulties at any time), and only one child (4%) showed any persistence of difficulties in this area. Similarly, for the pro-social problems domain, the comparison group showed onset most commonly at age 4 (82% of those showing problems at any time) and very little persistence (N = 5, 15%).

**Fig. 3:**
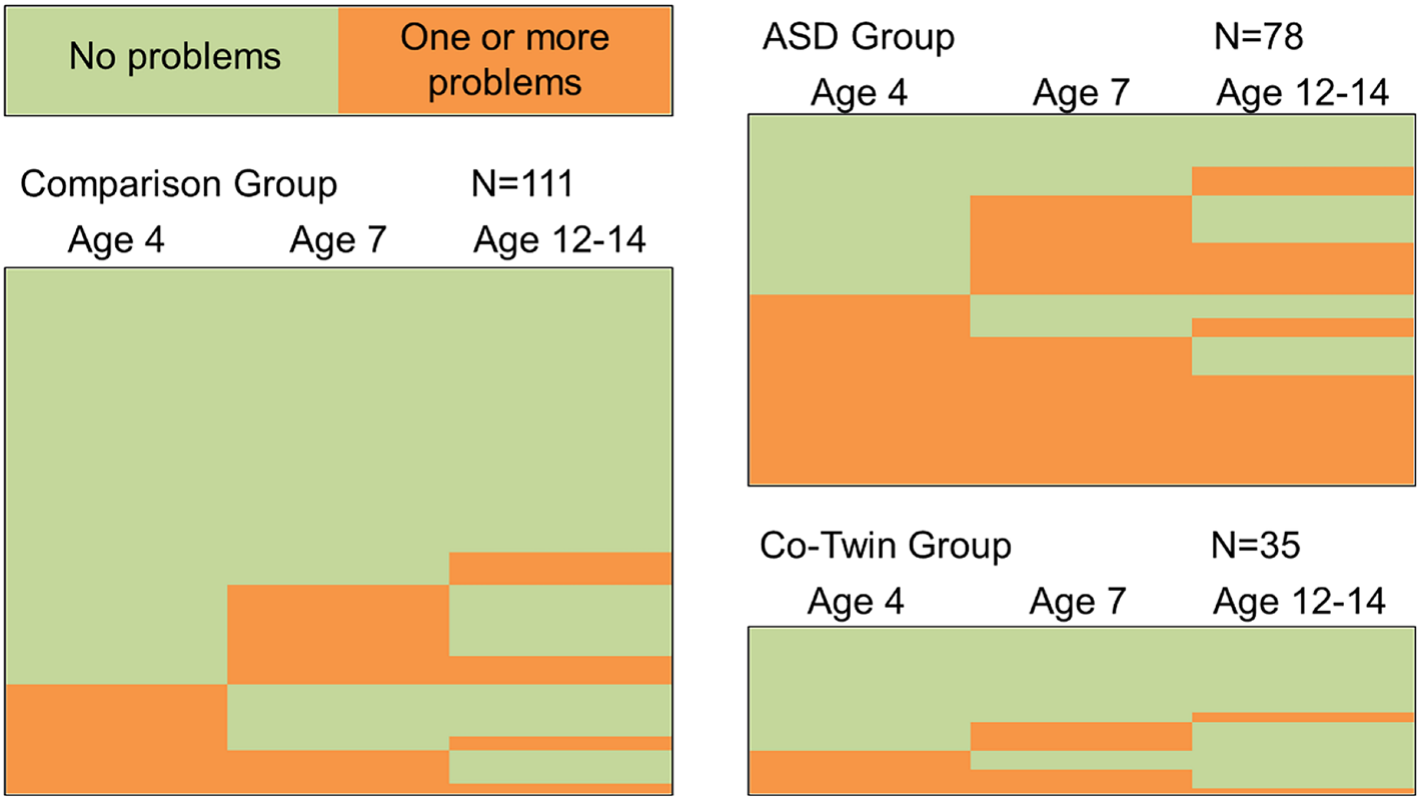
Longitudinal patterns of any above cut-off problems for SDQ emotional, conduct and hyperactivity combined (note each line represents one participant)

In addition to considering the three domains (emotional, conduct and hyperactivity) separately, an “any difficulties” analysis was also conducted to examine the longitudinal pattern of any above cut-off scores to allow for changes in the manifestation of problems over time. Figure 3 shows the pattern of results when all difficulties are considered. As can be seen, the ASD group showed far higher levels of difficulties over time; when any problem at any time point is considered, the ASD group showed significantly higher rates of problems than the co-twin and comparison groups; χ2 (2, N = 224) = 32.91, p < 0.001, V = 0.38. Of the 40 ASD children at age 4 showing above cut-off ratings in any of the three domains, 35 (88%) went on to have problems at one or more of the later time points. Conversely, of the 38 ASD children with no problems at age 4, 27 (71% of that group) went on to have problems at either age 7 or the SR Study time point (c. 13 years). The co-twin and comparison groups showed far higher levels of no problems and offset of difficulties over time. For the co-twins, of the 26 who had no problems at age 4, only 8 (31% of that group) went on to develop difficulties over time. Similarly, for the comparison group, of the 88 with no difficulties at age 4, only 28 (32% of that group) showed onset at the later time points.

## Discussion

The current study aimed to investigate the rates and types of co-occurring mental health difficulties in a population-based sample of twins covering the full autism spectrum. Five domains of possible difficulties were examined (emotional, conduct, hyperactivity, peer problems and pro-social difficulties), as measured by parent-report on the SDQ. ASD twins showed significantly more problems in all areas, when compared with their non-ASD co-twins or comparison twins selected for low ASD traits. The ASD group showed significantly higher domain scores and more difficulties that exceeded suggested cut-offs even in areas with no symptom overlap with autism (SDQ emotional, hyperactivity and conduct domains), while the comparison and co-twin samples did not differ. These findings support the previous research on comorbid difficulties in ASD, which has largely come from clinic-based samples and complement the work on subthreshold autistic traits in community samples—as well as replicating, with an independent sample and different measures, the findings of Simonoff et al. from the SNAP epidemiological study.

The current study also examined possible risk factors to see if the additional difficulties seen in the ASD group were related to aspects ordinarily found to be linked to childhood psychiatric conditions. The key finding from the analyses of risk factors was that for all three sample groups ASD/autism trait severity (as measured by ADI-R, ADOS-G or CAST) was the biggest contributor in terms of the SDQ domain scores. The higher participants scored in terms of ASD symptoms/traits the higher their SDQ scores were – and this was not only true for peer problems and the pro-social scale that clearly relate to ASD core symptomatology. It is important to note that for the ASD group both parent-rated ASD severity (ADI-R scores) and independent observer-rated severity (ADOS-G scores) showed the same pattern of relations to (parent-rated) SDQ scores, indicating that this association does not merely reflect common methods variance or a halo effect in reports from a single informant. When examined in terms of numbers of participants exceeding SDQ cut-off scores, ASD severity/traits again played a role, albeit reduced and mainly confined to the ASD group (potentially due to the smaller number passing cut-off in the other groups; although CAST was significantly linked to the pro-social domain for the comparison group). For the ASD group the link with ASD severity occurred for cut-off levels in hyperactivity and peer problems (see Tick et al., 2016, for twin analysis of the hyperactivity ASD link, which indicated that hyperactivity may show an effect on the identification of ASD). The present association between ASD severity/traits and co-occurring mental health is in line with findings from a large Swedish twin cohort study (Lundström et al., 2011) where even low levels of autistic like traits (as assessed using a DSM-IV based symptom algorithm) led to increased risk of additional mental health difficulties. The finding is, however, in contrast with Simonoff et al’s (2008) finding of no clear links between ASD severity and any of the domains of the CAPA in their population-based singleton sample. The differing findings could be related to the use of different tools to measure co-occurring difficulties, with the CAPA focusing more on clinical level difficulties, whilst the current study additionally included continuous SDQ scores, perhaps capturing more variance.

In addition to the effects of ASD severity, examination of associations with participant characteristics suggested some links between SES and conduct problems for the co-twins and comparison groups, which mirror previous findings for conduct type problems in typically developing populations (Murray & Farrington, 2010). IQ was found to be linked to hyperactivity, peer and pro-social difficulties for the ASD group continuous scores only, suggesting a role for IQ, but not one that extends into the realms of clinically significant cut-offs. The lack of any systematic link to IQ for any of the cut-off scores for any of the sample groups is perhaps the most surprising result, albeit one that has been reported previously in clinic (Brereton et al., 2006) and population-based samples (Simonoff et al., 2008). It is possible that the lack of any links between IQ and cut-off scores may be in part a result of a lack of power to detect these owing to the small number of non-ASD twins who scored within the clinically significant range. In addition to the IQ findings, there were no clear links to sex or age (at the SR Study time point, 12–14 years) for the cut-off scores, indicating that the significant problems shown by the ASD group were not related to factors ordinarily found to be influential in childhood difficulties. However, it should be noted that the small number of females included in the ASD sample may have reduced the power available to detect sex differences.

The prevalence of multiple difficulties was also of interest; previous research has indicated that 70% of a comparable population-based sample showed one additional problem area and up to 41% showed more than one (Simonoff et al., 2008). In the current study, 31% of the ASD group showed one domain of difficulty and as a whole the group exhibited far higher rates of multiple difficulties than either the comparison or co-twin groups, with an overall rate of 27% showing more than one domain rated above cut-off. It should be noted that the most recent, and revised, cut-off scores for the SDQ were used, which show improved specificity but are debatably more stringent than previous cut-offs.

The design of the current study allowed for an examination of the longitudinal course of the difficulties shown by the groups. At all-time points (ages 4, 7 and the most recent SR Study visit at age 12–14 years) the ASD group showed significantly higher scores for all SDQ domains and significantly higher rates of difficulties past cut-off. Change over time was investigated and, whilst the comparison and co-twins showed fluctuation and often decrease over time, the ASD group as a whole showed significant increases in terms of those scoring above cut-off for emotional, hyperactivity and peer problems. Previous research has implicated adolescence as a key time for the emergence of psychiatric problems in the general population (Bohman & Sigvardsson, 1980; Brodzinsky, 1993; Verhulst & Versluis-Den Bieman, 1995), however, in our ASD group onset of difficulties and persistence from ages 4 or 7 was common for all domains except peer and pro-social problems which showed higher rates at the later time point, which could reflect these aspects becoming of greater concern to parents as their children enter adolescence. It should be noted that the earlier time points may have underestimated the levels of difficulties for those with ASD as not all families in the ASD group took part at these times, potentially due to increased burden. The current findings support those of Simonoff et al. (2013) reporting persistence and domain specificity from childhood to early adolescence for additional difficulties in their population-based singleton sample.

### Limitations

The current study had several strengths, including a relatively large, population-based sample, assessed with standardised tools over several time-points, but of course there are also limitations to consider. Larger sample size would have allowed a more detailed examination of the difficulties shown in relation to subgroups within ASD, particularly by sex. Another limitation is the inclusion of only single-informant (parent) rated measures of mental health problems. It is possible that parental perceptions do not capture all aspects of young peoples’ functioning; for example, parents may under-estimate the difficulties of their non-ASD co-twins. We did collect self-ratings where possible, but reporting these would necessarily limit the sample to those autistic individuals without additional intellectual disability or language disorder. Future studies should include multiple informants (e.g., teachers, parents, self) where possible. In terms of the need for multiple informants, it is a strength of the study that both the ADI-R and ADOS-G were available for the ASD group, providing both the parent perspective and that of an independent observer. The inclusion of longitudinal analyses required the narrowing of the main sample to include only those with data at all three time points. Analyses revealed that this reduction of the sample may have led to the removal of some of the lower IQ individuals (some of whom were not included in earlier time points), this is therefore a limitation of this approach, and it should be noted when considering the findings for the longitudinal analyses. A final limitation is that the use of the SDQ limited the current study in terms of the areas of psychopathology that could be examined; future research could include more in-depth analysis of mental health and/or include broader areas such as sleep and eating difficulties.

## Conclusions

In summary, the results of the current study support previous research findings of high levels of comorbid difficulties for those with ASD. The population-based design and the use of standardised measures allow a clearer understanding of the rates and the nature of those difficulties for young people across the full spectrum of ASD. Those with ASD were far more likely to have marked and significant difficulties across all SDQ domains, broadly mirroring what has been found in previous research. Additionally, the ASD sample were more likely to have multiple problems above cut-off. The extent of ASD symptoms/traits (by parental report/observer ratings on gold-standard instruments) was the factor most strongly associated with SDQ ratings, for all three sample groups. The longitudinal results indicate that the additional problems for the ASD group can be different in terms of onset and more persistent than for the general population, showing a different longitudinal pattern to those exhibited by the comparison sample. These findings, together with the lack of links to non-ASD related risk factors such as SES, suggest that the profile of problems shown may be different in young people with versus without ASD. The very high rates of difficulties shown by the ASD group indicate that comorbidity is a serious issue and more evidence concerning the nature of comorbid difficulties is needed to help develop and target interventions for those with ASD.

## Supplementary Information

Below is the link to the electronic supplementary material.

Supplementary file1 (DOCX 33 kb)
