# Supplementary material for: Autism Spectrum Disorder and Mental Health Problems: Patterns of Difficulties and Longitudinal Trajectories in a Population-Based Twin Sample
Source: J Autism Dev Disord. 2021 Apr 17;52(3):1077–91. doi: 10.1007/s10803-021-05006-8 (PMC8854265; doi:10.1007/s10803-021-05006-8)
Supplement: Supplementary file 1 — Supplementary file1 (DOCX 33 kb) [file 10803_2021_5006_MOESM1_ESM.docx]

**Autism Spectrum Disorder and Mental Health Problems: Patterns of Difficulties and Longitudinal Trajectories in a Population-Based Twin Sample**

**Appendix 1:** **SDQ cut-off scoring criteria**

| **SDQ Domain** | **Close to average** | **Slightly raised (slightly lowered)** | **High (low)** | **Very high (very low)** |
| --- | --- | --- | --- | --- |
| Emotional | 0-3 | 4 | 5-6 | 7-10 |
| Conduct | 0-2 | 3 | 4-5 | 6-10 |
| Hyperactivity | 0-5 | 6-7 | 8 | 9-10 |
| Peer | 0-2 | 3 | 4 | 5-10 |
| Prosocial | 8-10 | 7 | 6 | 0-5 |

**Appendix 2: Normality data for SDQ scores**

| **SDQ Domain** | **Skewness** | **Kurtosis** | **Shapiro-Wilk** |
| --- | --- | --- | --- |
| Emotional | 0.94 | 0.28 | .89, *p<.001* |
| Conduct | 1.74 | 4.38 | .81, *p<.001* |
| Hyperactivity | 0.47 | -0.80 | .94, *p<.001* |
| Peer | 0.89 | -0.36 | .84, *p<.001* |
| Prosocial | -1.10 | 0.54 | .86, *p<.001* |

**Appendix 3: Full statistical details**

Table 3a: Post hoc Bonferroni corrected Dunn tests for Kruskal Wallis group comparisons of SDQ scores

| **SDQ Domain** | **Sample group comparison** | **Post hoc Bonferroni Dunn test result** |
| --- | --- | --- |
| Emotional | ASD and co-twins | *p<.001* |
|  | ASD and comparison | *p<.001* |
|  | Co-twins and comparison | *p=1.00* |
| Conduct | ASD and co-twins | *P=.001* |
|  | ASD and comparison | *P=.005* |
|  | Co-twins and comparison | *p=.58* |
| Hyperactivity | ASD and co-twins | *p<.001* |
|  | ASD and comparison | *p<.001* |
|  | Co-twins and comparison | *p=.50* |
| Peer | ASD and co-twins | *p<.001* |
|  | ASD and comparison | *p<.001* |
|  | Co-twins and comparison | *p=1.00* |
| Prosocial | ASD and co-twins | *p<.001* |
|  | ASD and comparison | *p<.001* |
|  | Co-twins and comparison | *p=1.00* |

Table 3b: ASD and comparison groups post hoc chi-square results for SDQ cut-off scores

| **SDQ Domain** | **Chi-square result** |
| --- | --- |
| Emotional | χ 2 (1, N=279) = 35.91, *p*<.001, *V* = .36 |
| Conduct | χ 2 (1, N=279) = 16.93, *p*<.001, *V* = .25 |
| Hyperactivity | χ 2 (1, N=279) = 35.87, *p*<.001, *V* = .36 |
| Peer | χ 2 (1, N=279) = 142.02, *p*<.001, *V* = .71 |
| Prosocial | χ 2 (1, N=279) = 59.80, *p*<.001, *V* = .46 |

Table 3c: ASD and co-twin groups post hoc chi-square results for SDQ cut-off scores

| **SDQ Domain** | **Chi-square result** |
| --- | --- |
| Emotional | χ 2 (1, N=190) = 20.77, *p*<.001, *V* = .33 |
| Conduct | χ 2 (1, N=190) = 6.87, *p=.009*, *V* = .19 |
| Hyperactivity | χ 2 (1, N=190) = 15.46, *p*<.001, *V* = .29 |
| Peer | χ 2 (1, N=190) = 72.42, *p*<.001, *V* = .62 |
| Prosocial | χ 2 (1, N=190) = 26.53, *p*<.001, *V* = .37 |

Table 3d: Co-twin and comparison groups post hoc chi-square results for SDQ cut-off scores

| **SDQ Domain** | **Chi-square result** |
| --- | --- |
| Emotional | χ 2 (1, N=199) = .47, *p=.49*, *V* = .05 |
| Conduct | χ 2 (1, N=199) = .07, *p=.79*, *V* = .02 |
| Hyperactivity | χ 2 (1, N=199) = .03, *p=.86*, *V* = .01 |
| Peer | χ 2 (1, N=199) = .15, *p=.70*, *V* = .03 |
| Prosocial | χ 2 (1, N=199) = .22, *p=.64*, *V* = .03 |

Table 3e: Full correlation details for risk factors for the ASD group

| **SDQ Domain** | **Age** | **Composite IQ** | **SES** | **ADOS-CSS** | **ADI-R** |
| --- | --- | --- | --- | --- | --- |
| Emotional | -.06  p=.491 | -.06  p=.488 | -.12  p= .287 | -.02  p=.862 | -.01  p=.917 |
| Conduct | .05  p=.604 | -.15  p=.109 | -.16  p=.145 | .06  p=.525 | .14  p=.120 |
| Hyperactivity | -.01  p=.957 | -.22  p=.017 | -.10  p=.382 | .19  p=.036 | .34  p<.001 |
| Peer | .09  p=.308 | -.24  p=.008 | -.02  p=.849 | .32  p<.001 | .44  p<.001 |
| Pro-social | .06  p=.538 | .18  p=.047 | -.18  p=.102 | -.17  p=.057 | -.43  p<.001 |

Table 3f: Full correlation details for risk factors for the co-twin group

| **SDQ Domain** | **Age** | **Composite IQ** | **SES** | **ADOS-CSS** | **ADI-R** |
| --- | --- | --- | --- | --- | --- |
| Emotional | -.12  p=.395 | -.13  p=.363 | -.21  p= .174 | .22  p=.106 | .32  p=.020 |
| Conduct | .02  p=.916 | -.15  p=.295 | -.55  p<.001 | .01  p=.976 | .17  p=.232 |
| Hyperactivity | -.04  p=.755 | -.29  p=.033 | -.49  p=.001 | .01  p=.973 | .26  p=.063 |
| Peer | -.03  p=.849 | -.23  p=.096 | -.23  p=.135 | .02  p=.884 | .33  p=.015 |
| Pro-social | .03  p=.819 | .01  p=.993 | .13  p=.426 | -.10  p=.457 | -.18  p=.206 |

Table 3g: Full correlation details for risk factors for the comparison group

| **SDQ Domain** | **Age** | **Composite IQ** | **SES** | **CAST** |
| --- | --- | --- | --- | --- |
| Emotional | -.02  p=.837 | -.14  p=.100 | -.13  p= .130 | .16  p=.060 |
| Conduct | .22  p=.007 | -.19  p=.021 | -.17  p=.044 | .35  p<.001 |
| Hyperactivity | .15  p=.072 | -.35  p<.001 | -.24  p=.005 | .32  p<.001 |
| Peer | .19  p=.020 | -.21  p=.013 | -.06  p=.514 | .18  p=.039 |
| Pro-social | -.12  p=.140 | .11  p=.187 | .16  p=.058 | -.23  p=.005 |

Table 3h: Kruskal-Wallis and post hoc Bonferroni corrected Dunn tests for age 4 SDQ scores

| **SDQ Domain** | **Kruskal-Wallis result** | **Post hoc Bonferroni Dunn test result** | |
| --- | --- | --- | --- |
| Emotional | χ 2 (2) = 7.31, *p=.026*, ƞ^2^ = 0.02 | ASD and comparison | *p=.026* |
|  |  | ASD and co-twin | *p=.26* |
|  |  | Comparison and co-twin | *p=1.00* |
|  |  |  |  |
| Conduct | χ 2 (2) = 14.54, *p=*.001, ƞ^2^ = 0.06 | ASD and comparison | *p=.002* |
|  |  | ASD and co-twin | *p=.008* |
|  |  | Comparison and co-twin | *p=1.00* |
|  |  |  |  |
| Hyperactivity | χ 2 (2) = 19.38, *p*<.001, ƞ^2^ = 0.08 | ASD and comparison | *p=.002* |
|  |  | ASD and co-twin | *p<.001* |
|  |  | Comparison and co-twin | *p=.40* |
|  |  |  |  |
| Peer | χ 2 (2) = 56.32, *p*<.001, ƞ^2^ = 0.25 | ASD and comparison | *p<.001* |
|  |  | ASD and co-twin | *p<.001* |
|  |  | Comparison and co-twin | *p=1.00* |
|  |  |  |  |
| Prosocial | χ 2 (2) = 12.81, *p=.002*, ƞ^2^ = 0.05 | ASD and comparison | *p=.008* |
|  |  | ASD and co-twin | *p=.007* |
|  |  | Comparison and co-twin | *p=1.00* |
|  |  |  |  |

Table 3i: Kruskal-Wallis and post hoc Bonferroni corrected Dunn tests for age 7 SDQ scores

| **SDQ Domain** | **Kruskal-Wallis result** | **Post hoc Bonferroni Dunn test result** | |
| --- | --- | --- | --- |
| Emotional | χ 2 (2) = 26.90, *p*<.001, ƞ^2^ = 0.11 | ASD and comparison | *p<.001* |
|  |  | ASD and co-twin | *p=.118* |
|  |  | Comparison and co-twin | *p=.221* |
|  |  |  |  |
| Conduct | χ 2 (2) = 22.34, *p*<.001, ƞ^2^ = 0.09 | ASD and comparison | *p<.001* |
|  |  | ASD and co-twin | *p=.006* |
|  |  | Comparison and co-twin | *p=1.00* |
|  |  |  |  |
| Hyperactivity | χ 2 (2) = 49.69, *p*<.001, ƞ^2^ = 0.22 | ASD and comparison | *p<.001* |
|  |  | ASD and co-twin | *p<.001* |
|  |  | Comparison and co-twin | *p=1.00* |
|  |  |  |  |
| Peer | χ 2 (2) = 84.13, *p*<.001, ƞ^2^ = 0.37 | ASD and comparison | *p<.001* |
|  |  | ASD and co-twin | *p<.001* |
|  |  | Comparison and co-twin | *p=1.00* |
|  |  |  |  |
| Prosocial | χ 2 (2) = 28.30, *p*<.001, ƞ^2^ = 0.12 | ASD and comparison | *p<.001* |
|  |  | ASD and co-twin | *p=.001* |
|  |  | Comparison and co-twin | *p=1.00* |
|  |  |  |  |

Table 3j: Chi-square results for age 4 SDQ cut-off scores

| **SDQ Domain** | **Overall Chi-square result** | **Post hoc chi-square results** | |
| --- | --- | --- | --- |
| Emotional | χ 2 (2, N=224) = 12.12, *p=.002*, *V* = .23 | ASD and comparison | χ 2 (1, N=213) = 10.88, *p=.001*, *V* = .23 |
|  |  | ASD and co-twin | χ 2 (1, N=137) = 2.10, *p=.15*, *V* = .12 |
|  |  | Comparison and co-twin | χ 2 (1, N=158) = 1.09, *p=.30*, *V* = .08 |
|  |  |  |  |
| Conduct | χ 2 (2, N=224) = 9.25, *p=.01*, *V* = .20 | ASD and comparison | χ 2 (1, N=213) = 10.53, *p=.001*, *V* = .22 |
|  |  | ASD and co-twin | χ 2 (1, N=137) = 3.73, *p=.05*, *V* = .17 |
|  |  | Comparison and co-twin | χ 2 (1, N=158) = .15, *p=.70*, *V* = .03 |
|  |  |  |  |
| Hyperactivity | χ 2 (2, N=224) = 25.32, *p<.001*, *V* = .34 | ASD and comparison | χ 2 (1, N=213) = 21.51, *p<.001*, *V* = .32 |
|  |  | ASD and co-twin | χ 2 (1, N=137) = 9.80, *p=.002*, *V* = .27 |
|  |  | Comparison and co-twin | χ 2 (1, N=158) = .10, *p=.76*, *V* = .03 |
|  |  |  |  |
| Peer | χ 2 (2, N=224) = 55.09, *p<.001*, *V* = .50 | ASD and comparison | χ 2 (1, N=213) = 56.95, *p<.001*, *V* = .52 |
|  |  | ASD and co-twin | χ 2 (1, N=137) = 28.31, *p<.001*, *V* = .46 |
|  |  | Comparison and co-twin | χ 2 (1, N=158) = .06, *p=.81*, *V* = .02 |
|  |  |  |  |
| Prosocial | χ 2 (2, N=224) = 12.03, *p=.002*, *V* = .23 | ASD and comparison | χ 2 (1, N=213) = 10.42, *p=.001*, *V* = .22 |
|  |  | ASD and co-twin | χ 2 (1, N=137) = 7.47, *p=.006,* *V* = .23 |
|  |  | Comparison and co-twin | χ 2 (1, N=158) = .22, *p=.64*, *V* = .04 |
|  |  |  |  |

Table 3k: Chi-square results for age 7 SDQ cut-off scores

| **SDQ Domain** | **Overall Chi-square result** | **Post hoc chi-square results** | |
| --- | --- | --- | --- |
| Emotional | χ 2 (2, N=224) = 20.17, *p<.001*, *V* = .30 | ASD and comparison | χ 2 (1, N=222) = 20.11, *p<.001*, *V* = .30 |
|  |  | ASD and co-twin | χ 2 (1, N=134) = 6.34, *p=.012*, *V* = .22 |
|  |  | Comparison and co-twin | χ 2 (1, N=172) = .47, *p=.49*, *V* = .05 |
|  |  |  |  |
| Conduct | χ 2 (2, N=224) = 29.24, *p<.001*, *V* = .36 | ASD and comparison | χ 2 (1, N=222) = 35.23, *p<.001*, *V* = .40 |
|  |  | ASD and co-twin | χ 2 (1, N=134) = 10.90, *p=.001*, *V* = .29 |
|  |  | Comparison and co-twin | χ 2 (1, N=172) = .87, *p=.35*, *V* = .07 |
|  |  |  |  |
| Hyperactivity | χ 2 (2, N=224) = 41.37, *p<.001*, *V* = .43 | ASD and comparison | χ 2 (1, N=222) = 31.15, *p<.001*, *V* = .38 |
|  |  | ASD and co-twin | χ 2 (1, N=134) = 15.88, *p<.001*, *V* = .34 |
|  |  | Comparison and co-twin | χ 2 (1, N=172) = .13, *p=.72*, *V* = .03 |
|  |  |  |  |
| Peer | χ 2 (2, N=224) = 55.83, *p<.001*, *V* = .50 | ASD and comparison | χ 2 (1, N=222) = 51.12, *p<.001*, *V* = .48 |
|  |  | ASD and co-twin | χ 2 (1, N=134) = 22.83, *p<.001*, *V* = .41 |
|  |  | Comparison and co-twin | χ 2 (1, N=172) = .01, *p=.91*, *V* = .01 |
|  |  |  |  |
| Prosocial | χ 2 (2, N=224) = 15.23, *p<.001*, *V* = .26 | ASD and comparison | χ 2 (1, N=222) = 23.22, *p<.001*, *V* = .32 |
|  |  | ASD and co-twin | χ 2 (1, N=134) = 11.88, *p=.001*, *V* = .30 |
|  |  | Comparison and co-twin | χ 2 (1, N=172) = .02, *p=.88*, *V* = .01 |
|  |  |  |  |
